# Behavioral Responses to Neural Circuit Control: Pitfalls and Possible Solutions

**DOI:** 10.1523/ENEURO.0223-21.2021

**Published:** 2021-07-08

**Authors:** Oscar Solis

**Affiliations:** National Institute on Drug Abuse Intramural Research Program, Baltimore, MD 21224

## Abstract

**Highlighted Research Paper:** Lewis AS, Calipari ES, Siciliano CA (2021) Toward Standardized Guidelines for Investigating Neural Circuit Control of Behavior in Animal Research.

One of the fundamental goals of neuroscience is to gain a better understanding of the brain’s role in behavior (Kandel et al., 2013). In the last two decades, the development of new technologies has significantly advanced our knowledge about how neural circuits generate/regulate behavior. Technologies such as optogenetics and chemogenetics, in combination with pharmacological approaches, lesions, gene mutations, and/or imaging, have helped delineate the involvement of specific neurons or anatomic pathways in different behaviors (Kim et al., 2017; Boehm et al., 2021). This has facilitated the attribution of causal relationships between discrete neuronal circuits and behavior. Nevertheless, despite the associated scientific progress, the application of such technologies in neuroscience is not exempt of difficulties or controversy. Reproducibility (or its lack thereof) is a common problem in behavioral experimentation and therefore of fundamental importance and necessity to identify strategies that promote it.

Lewis et al. (2021) set the focus on this topic and provide a discussion about common pitfalls that can lead to misinterpretation of behavioral outcomes during neuronal manipulations. The authors propose several guidelines that could be adopted to improve rigor, reproducibility, and interpretation of behavioral experimentation. To ensure that the tools are being used correctly, strategies need to be carefully planned and executed depending on the scientific questions that need to be answered.

In behavioral neuroscience, there is a tendency to develop automated tasks (Schaefer and Claridge-Chang, 2012). Automated tests are attractive because they can quantify behavior in an unbiased fashion. However, automated scoring is not free from experimental errors that could lead to errors in the interpretation of the results. For instance, if an automated system registers a behavioral response (e.g., the number of lever presses performed by an animal) after manipulation of a discrete circuit, one could miss the various factors that are involved in generating this response, such as motor behavior, exploratory behavior, arousal, or even sleep induction. To address this, Lewis et al. (2021) propose the use of a webcam for recording behavior during a specific task. By using this approach, multiple researchers can observe the experiment repeatedly, play it in back in slow motion, and for publication purposes, the submission of the footage can be archived in online repositories.

Over the last years, a growing body of research has associated the activity of discrete neurons and circuits with specific behavioral outcomes. However, in certain cases, these interpretations have been based on studying behavior using a single paradigm or procedure. Given the complexity of behavior and the multitude of different factors that can affect animal responses, researchers should consider testing additional paradigms or procedures when designing behavioral experiments to lend further support to the interpretation of their initial data. Another important point emphasized by Lewis et al. (2021) involved the overgeneralization of study conclusions. Here, they specifically highlight the importance of describing the behavior itself, placing key emphasis on what is observed during the experiment, so that the conclusions can be fully supported by the data.

The field of neuroscience has adopted recent technologies with great success and more exciting and sophisticated technologies are on the horizon. However, these tools, and their application to the study of complex behavior, are continuously evolving. For this reason, Lewis et al. (2021) propose the need to standardize behavioral testing to reduce the probability of replication failure. In addition, Lewis et al. (2021) note several considerations for future research, including: observation and visual recording of behavioral assays, reporting data in more precise terms that are separate from speculation, reducing the emphasis on defining specificity, and manipulating circuits across a wide range of procedures. Notably, careful consideration should be given to biological and technical variables that can alter the results and their interpretation. Following such practices will ensure greater reproducibility and generalization in the process of attributing causality between discrete neuronal circuit perturbation and behavior.

## Synthesis

Reviewing Editor: Christophe Bernard, INSERM & Institut de Neurosciences des Systèmes
